# ISE/ISHNE expert consensus statement on the ECG diagnosis of left ventricular hypertrophy: The change of the paradigm

**DOI:** 10.1111/anec.13097

**Published:** 2023-11-24

**Authors:** Ljuba Bacharova, Philippe Chevalier, Bulent Gorenek, Christian Jons, Yi‐Gang Li, Emanuela T. Locati, Maren Maanja, Andrés Ricardo Pérez‐Riera, Pyotr G. Platonov, Antonio Luiz Pinho Ribeiro, Douglas Schocken, Elsayed Z. Soliman, Jana Svehlikova, Larisa G. Tereshchenko, Martin Ugander, Niraj Varma, Zaklyazminskaya Elena, Takanori Ikeda

**Affiliations:** ^1^ International Laser Center CVTI Bratislava Slovak Republic; ^2^ Neuromyogene Institute Claude Bernard University Villeurbanne France; ^3^ Service de Rythmologie Hospices Civils de Lyon Lyon France; ^4^ Eskisehir Osmangazi University Cardiology Department Eskisehir Turkey; ^5^ Department of Cardiology Rigshospitalet, Copenhagen University Hospital Copenhagen Denmark; ^6^ Department of Cardiology, Xinhua Hospital Shanghai Jiao Tong University School of Medicine Shanghai China; ^7^ Department of Arrhythmology and Electrophysiology IRCCS Policlinico San Donato Milano Italy; ^8^ Department of Clinical Physiology Karolinska University Hospital, and Karolinska Institutet Stockholm Sweden; ^9^ Uni (Uninove) Mauá SP Brazil; ^10^ Centro Universitário FMABC Santo André SP Brazil; ^11^ Department of Cardiology, Clinical Sciences Lund University Lund Sweden; ^12^ Internal Medicine, Faculdade de Medicina da Universidade Federal de Minas Gerais Belo Horizonte Brazil; ^13^ Telehealth Center, Hospital das Clínicas da Universidade Federal de Minas Gerais Belo Horizonte Brazil; ^14^ Division of Cardiology, Department of Medicine Duke University Medical Center Durham North Carolina USA; ^15^ Section on Cardiovascular Medicine, Department of Medicine, Epidemiological Cardiology Research Center Wake Forest University School of Medicine Winston‐Salem North Carolina USA; ^16^ Institute of Measurement Sciences, Slovak Academy of Sciences Bratislava Slovak Republic; ^17^ Department of Quantitative Health Sciences Lerner Research Institute, Cleveland Clinic Cleveland Ohio USA; ^18^ Faculty of Medicine and Health The University of Sydney Sydney New South Wales Australia; ^19^ Department of Clinical Physiology Karolinska Institute Stockholm Sweden; ^20^ Cardiac Pacing & Electrophysiology Heart and Vascular Institute, Cleveland Clinic Cleveland Ohio USA; ^21^ Medical Genetics Laboratory Petrovsky National Research Centre of Surgery Moscow Russia; ^22^ Toho University Faculty of Medicine Tokyo Japan

**Keywords:** ECG, left ventricular hypertrophy, new paradigm

## Abstract

The ECG diagnosis of LVH is predominantly based on the QRS voltage criteria. The classical paradigm postulates that the increased left ventricular mass generates a stronger electrical field, increasing the leftward and posterior QRS forces, reflected in the augmented QRS amplitude. However, the low sensitivity of voltage criteria has been repeatedly documented. We discuss possible reasons for this shortcoming and proposal of a new paradigm. The theoretical background for voltage measured at the body surface is defined by the *solid angle theorem*, which relates the measured voltage to spatial and non‐spatial determinants. The spatial determinants are represented by the extent of the activation front and the distance of the recording electrodes. The non‐spatial determinants comprise electrical characteristics of the myocardium, which are comparatively neglected in the interpretation of the QRS patterns. Various clinical conditions are associated with LVH. These conditions produce considerable diversity of electrical properties alterations thereby modifying the resultant QRS patterns. The spectrum of QRS patterns observed in LVH patients is quite broad, including also left axis deviation, left anterior fascicular block, incomplete and complete left bundle branch blocks, Q waves, and fragmented QRS. Importantly, the QRS complex can be within normal limits. The new paradigm stresses the electrophysiological background in interpreting QRS changes, i.e., the effect of the non‐spatial determinants. This postulates that the role of ECG is not to estimate LV size in LVH, but to understand and decode the underlying electrical processes, which are crucial in relation to cardiovascular risk assessment.

## Graphical abstract

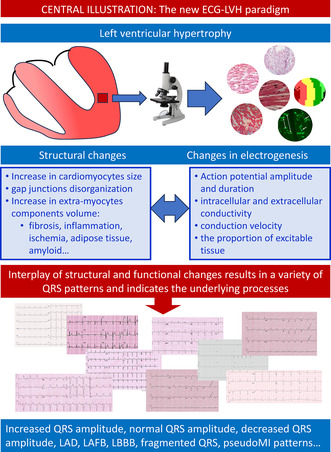

## 1 INTRODUCTION

The electrocardiographic diagnosis of left ventricular hypertrophy (ECG‐LVH) has a long history. The ECG changes in a patient with left ventricular hypertrophy (LVH) were described 117 years ago by Einthoven in 1906 (Einthoven, 1957). He drew attention to the distinctive finding—the increased QRS amplitude in the “left hand to left foot lead” (i.e., lead III). Since then, the increased QRS amplitude has been considered pathognomonic sign of LVH.

The diagnosis of LVH is clinically important. LVH changes the structure and function of the heart; it is associated with a variety of clinical conditions and can lead to serious complications. The ECG is economically and clinically the most accessible diagnostic and screening tool.

**FIGURE 1:**
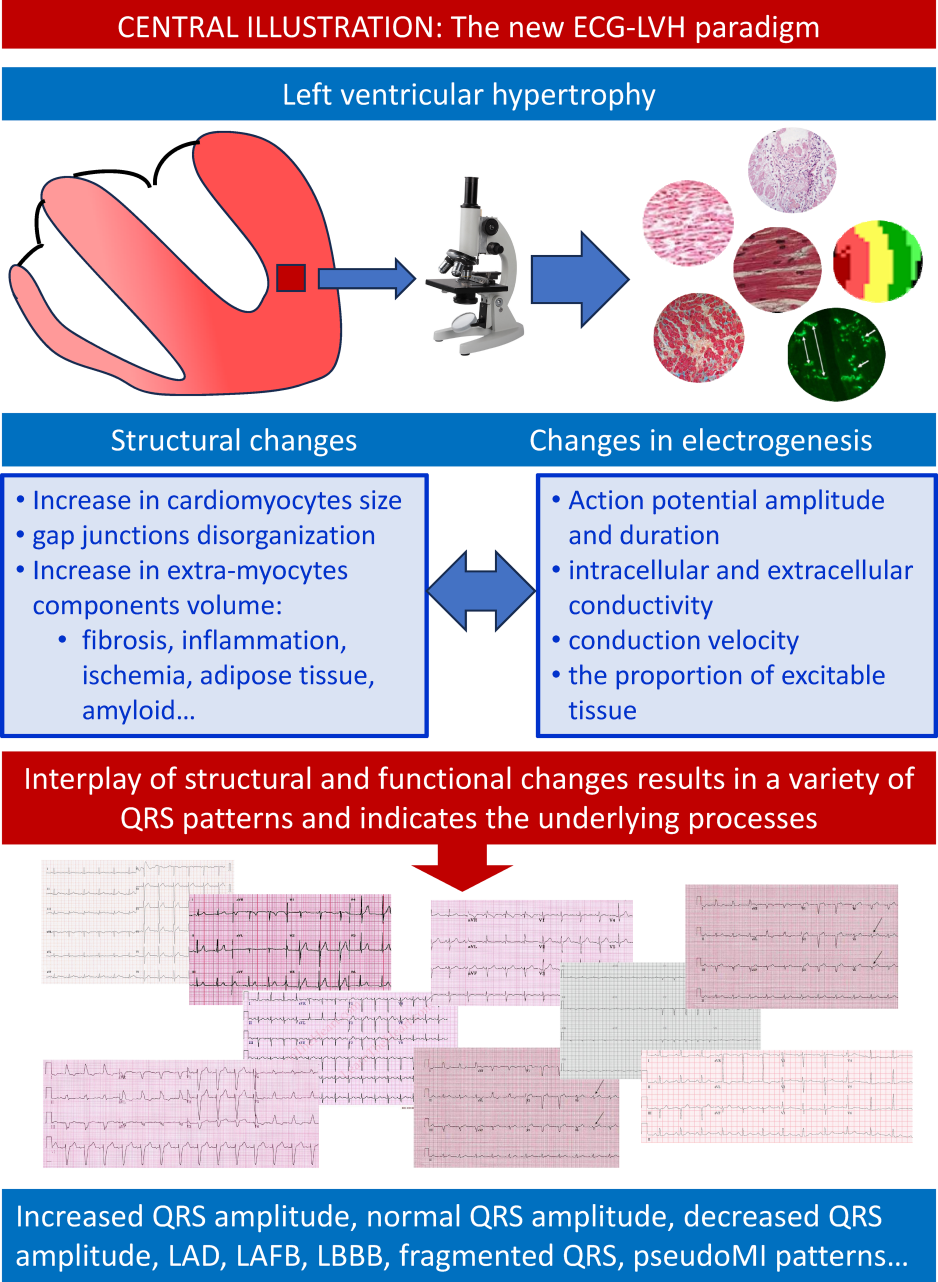
Central illustration: The new ECG‐LVH paradigm. LAD, left axis deviation; LAFB, left anterior fascicular block; LBBB, left bundle branch block.

Numerous diagnostic criteria for LVH have been proposed over the years. However, all suffer from low sensitivity in LVH detection. At the same time, increasing sophistication of imaging technologies allowing assessment of the anatomy and structure of the heart have positioned these as methods of choice to detect LVH. These factors have diminished the interest in ECG in patients with LVH. However, the ECG imparts unique information on the electric field associated with LVH. Increased QRS amplitude (Adam Leigh et al., 2016; Zhang et al., 2020), in the presence of LVH, are significant independent cardiovascular risk factors (Bacharova et al., 2015; Pelliccia et al., 2023). Therefore, the electrical characteristics of the heart depicted by the ECG provide valuable information adjunctive to imaging and cannot be neglected. The aim of this statement from ECG societies is to critically appraise the classical diagnostic paradigm of ECG‐LVH, to revise the understanding of ECG's role in LVH diagnosis according to the current knowledge and outline the directions for future research (Figure 1).

## 2 LVH DEFINITION

Hypertrophy is basically defined as the increase in the volume of an organ or tissue due to the enlargement of its component cells. Accordingly, left ventricular hypertrophy should be basically defined as the increase in the volume of the left ventricle or its part due to the enlargement of its component cells—implicitly cardiomyocytes. The pathophysiological understanding of LVH extends this definition and characterized LVH as the increased growth of adult cardiomyocytes and associated interstitium remodeling, including increased interstitial fibrosis, apoptosis, and abnormal cardiac function (Heuther & McCance, 2017). This complex process also includes an excess amount of subcellular extracellular components such as collagen, amyloid proteins, or inflammatory/edematous processes (Broberg et al., 2010). Using CMR, the extracellular volume fraction (ECV) can be quantified as a surrogate for diffuse myocardial fibrosis (Miller et al., 2013). Also, the combination of ECV and LVM can be used to differentiate quantitatively the intracellular and extracellular components of changes in LVM (Maestrini et al., 2014). In clinical practice, the term ‘LVH’ is more loosely applied and frequently mutually interchanged by the term ‘enlargement’. This enlargement could be mass, chamber dilatation, wall thickness, interstitial components, or their combination.

## 3 CLINICAL LVH

LVH is defined as increased left ventricular mass (LVM). Its measurement depends on the method used for LVH estimation. An autopsy can directly measure the size and weight of the left ventricle and defines LVH as the weight of LVM exceeding the arbitrary upper normal limit (Dadgar & Tyagi, 1979). Non‐invasive imaging methods—allow imaging of the LV size and estimate the LV volume. The product of the estimated LV volume and the myocardial density of 1.05 g/mL is used for calculating LVM with echocardiography. CMR may be more precise. To decrease the effect of different body sizes, the estimated LVM is indexed to the body weight or surface. LVH is then defined as LVM exceeding the upper normal limits.

Multiple cardiovascular conditions are associated with LVH and cause distinct hypertrophy patterns, myocardial structural alterations, and ECG manifestations. LVH may occur as an adaptation process in response to pressure or volume overload, or be intrinsic (congenital/genetic). A few examples are listed.

## 4 PATHOLOGY

LVH in response to overload is seen in hypertension, congenital heart disease, and valvular defects, as well as post‐infarction myocardial remodeling as the compensatory hypertrophy in the noninfarcted region (Pfeffer & Braunwald, 1990; Sharpe, 1992). Histology shows substantial structural changes, such as enlarged cardiomyocytes, gap junction remodeling, and extracellular matrix remodeling, including diffuse and localized fibrosis and areas of inflammation. The imbalance between the increased LVM and blood supply may lead to ischemia with additional subsequent damage of the myocardium. The changes in electrical properties include changes in action potential morphology, duration, and conduction velocity (CV) affecting electrical impulse propagation. LVH can also develop as a physiological response to exercise and pregnancy. However, aging, long‐term intensive exercise and sustained workload can lead to pathological hypertrophy with structural and functional manifestations.

Hypertrophic cardiomyopathy (HCM) is an autosomal dominant genetic disease (Zamorano et al., 2014). HCM is often asymmetric, and the most frequent location involves the basal interventricular septum causing left ventricular outflow tract obstruction at rest in about one‐third of the patients (Marian & Braunwald, 2017). In contrast to hypertrophy induced by increased workload, hypertrophy in HCM is characterized by myofibrillar disarray, fibrotic scars, and abnormal internal coronary vessels (Blauwet et al., 2009). The ECG findings of HCM include an increased QRS amplitude as well as pseudo‐myocardial infarction QRS patterns.

Obesity leads to LVH basically by two mechanisms: (1) eccentric hypertrophic remodeling due to increased cardiac output and wall stress imposed by this cavity dilatation; (2) concentric hypertrophy, which is related to insulin resistance, hyperleptinaemia, and myocardial steatosis (Alpert, 2001; Henry et al., 2023; Masaidi et al., 2009; Rider et al., 2011). The increase of LVM is conditioned not only by the growth of cardiomyocytes but as well by interstitial fat infiltration as well as the triglyceride accumulation in cardiomyocytes (Murdolo et al., 2015). The QRS amplitude is reported to be lower in obese subjects (Abergel et al., 1996). However, Eisenstein et al. (1982) showed that QRS voltage in obese patients was slightly lower than the voltage of the normal population, but the QRS amplitude decreased in a significant number of patients after weight loss. No differences in QRS amplitude between obese and lean subjects were also reported, while the leftward shift of the electric axis is a consistent finding (Bacharova et al., 2018; Kurisu et al., 2015). The changes in the myocardium are even more enhanced in metabolic syndrome patients, combining the effect of hypertension, obesity, and insulin resistance.

Cardiac amyloidosis (CA) is an infiltrative cardiomyopathy caused by the extracellular accumulation of amyloid in the extracellular space. Histology shows cardiomyocyte degeneration, the extent and pattern of interstitial amyloid deposits and vascular deposits, endomyocardial deposition, or fibrosis (Koutroumpakis et al., 2023; Larsen et al., 2016; Pucci et al., 2021). The QRS abnormalities in CA patients are heterogeneous. In spite that LVM is increased, i.e., LVH is diagnosed clinically, the proportion of the working myocardium with respect to the extracellular space is decreased, resulting in low QRS amplitude, but also pseudo‐infraction Q waves, as well as conduction disorders such as left or right bundle branch blocks and fascicular block are observed (Pericet‐Rodriguez et al., 2022). Other conditions with myocardial infiltration with similar QRS patterns include cardiac sarcoidosis, hemochromatosis, Danon disease, and Fabry's disease.

Heart failure (HF) refers to the final stage of LVH, when the heart fails to provide adequate perfusion of the systemic circulation by either systolic dysfunction, diastolic dysfunction, or both. This is beyond the scope of this statement. However, remodeling in HF is characterized by substantial anatomical and structural leading to LV dilation, with accompanying hypertrophied cardiomyocytes, apoptosis, local ischemia, and fibrosis. This may lead to increased LV mass. Together, these factors influence the electrical properties of LV. The characteristic ECG finding is the prolonged QRS duration (Murkofsky et al., 1997). QRS prolongation may also result from intraventricular conduction disorders, including the left bundle branch block (LBBB). In this context, adjustment of QRS duration for LV mass may better describe the conduction deficit that is treatable by CRT (Varma et al., 2017). Under certain conditions, the surface ECG pattern of “LBBB” may be misleading by implying a conduction problem in the left bundle branch, whereas it is generated by deterioration in myocardial conductivity, fundamental in HF. This distinction is of vital importance, especially in relation to the patient's indication for CRT.

Cardiac hypertrophy in the absence of pressure overload usually has a strong genetic component (Mayosi et al., 2008). Several multi‐systemic disorders have progressive cardiac hypertrophy as a part of their symptom complex (Pieroni et al., 2022). These disorders can be confused at the early stage with hypertrophic cardiomyopathy (HCM). HCM is sometimes used as a name for the group of disorders caused by genetic alterations in the components of contractile machinery. More than 60 genes are associated with isolated cardiac hypertrophy (Thomson et al., 2019). The strongest evidence for causality belongs to genes encoding 8 sarcomeric proteins (Ingles et al., 2019). The idea that different genetic types of HCM might have special features and, probably, treatment, was very promising. It was shown that the ultrasound phenotype of hypertrophy strongly depends on genetic background (Bos et al., 2009) and that mutations affecting thin components of the sarcomere may have a more “arrhythmic” phenotype than HCM‐caused thick filament disorders (Coppini et al., 2014; Keyt et al., 2022). Extensive research was performed to uncover genotype–phenotype correlation and incorporate particular mutations' role in predictive counseling, including sudden cardiac risk (SCD) estimation. There remains, however, a critical knowledge gap in our understanding of molecular mechanisms of sarcomeric HCM and ways to modulate its natural history. The link to ECG is a challenge and needs to be elucidated.

## 5 REFERENCE METHODS

Since LVH is defined as an increased LVM, it is diagnosed using cardiac imaging modalities such as echocardiography, cardiovascular magnetic resonance (CMR), or coronary computed tomography angiography (CCTA; Walker et al., 2016). These imaging modalities are also important tools for monitoring the progression of the disease and evaluating the effectiveness of therapeutic interventions. The choice of imaging method depends on the individual patient and the clinical scenario. Echocardiography is most readily available and often but not always less expensive, while CCTA and CMR can each provide more detailed and precise information on cardiac anatomy.

Echocardiography is a commonly used imaging method that uses high‐frequency sound waves to produce images of the heart. By measuring the ventricular wall's thickness and the ventricle's size, echocardiography can provide a reliable assessment of LVH with prognostic implications (Levy et al., 1990). In addition, echocardiography can also detect changes in the heart's function, such as decreased contractility and blood flow, which may be indicative of LVH.

Echocardiography offers several possibilities for LVM estimation: (1) M‐mode echocardiography uses a rough cubic formula for calculating the LV volume, and the final values of LV volume are overestimated; (2) 2D‐echocardiography uses an ellipsoid assumption for calculating the LV volume, the estimated volume can be overestimated as well as underestimated; (3) 3D‐echocardiography is without geometric assumption, the LV volume estimates are more precise (Chuang et al., 2000; Kusunose et al., 2013; Perdrix et al., 2011).

CMR does not expose the patient to ionizing radiation or require the use of intravenous contrast agents for quantifying LVM. Due to its excellent accuracy, precision, and reproducibility, CMR is the non‐invasive reference standard for assessing left ventricular mass, volumes, and systolic function (Grothues et al., 2002). CMR uses the short‐axis cine multi‐slices, sampling the ventricles from the atrioventricular ring to the apex. The derived volume is independent of geometric assumptions. This is the major advantage in estimating LVM compared to M‐mode and 2D echocardiography.

## 6 ELECTROPATHOLOGY OF HYPERTROPHIED MYOCARDIUM

Electrical properties of the hypertrophied myocardium vary depending on the type and severity of hypertrophy, as well as the degree of heterogeneity of structural alteration affecting impulse generation and propagation (Bacharova, 2007). Our understanding of electrical properties in LVH is to a large extent based on experimental and simulation studies, while data from human studies are scarce. While animal data are largely dependent on the experimental set‐up, the type of LVH model, and the species used, there is little doubt that hypertrophied myocardium exhibits altered electrophysiology, providing a substrate for reentry and risk of arrhythmias.

The factors underlying the development of electrical alterations in LVH include the following: (1) abnormal expression of potassium and calcium channels and altered calcium handling associated with prolongation of action potential duration (Botchway et al., 2003; Flenner et al., 2021; Kamei et al., 2007) and refractory period (Rials et al., 2001), (2) altered expression and distribution of gap junctions affecting the propagation of electrical impulses (Emdad et al., 2001; Peters, 1996), and (3) increased heterogeneity of myocardial electrical properties (Kashii & Imamura, 1976; McCrossan et al., 2004). Notably, electrical alterations were shown to be reversible upon resolution of LVH with complete normalization of dispersion of refractoriness, action potential duration and IK1 density (Rials et al., 1997; Rials et al., 2001).

Limited studies performed in humans with LVH, however, showed a more complex picture. Though most of the studies demonstrate delayed activation of ventricular myocardium in patients with increased myocardial mass, action potential duration and CV itself do not appear to be significantly affected (van Dam et al., 1972; Winterton et al., 1994).

## 7 THE CLASSICAL ECG‐LVH PARADIGM

The classical diagnostic paradigm postulates that the increased LVM results in a bigger activation wavefront which is reflected in the increased QRS amplitude on the surface ECG, e.g.,

“The increased voltage is attributed to one or more of the following factors: increased left ventricular mass, increased left ventricular surface, increased intracavitary blood volume, and close proximity of the enlarged ventricle to the chest wall.” “The increase in the left ventricular mass exaggerates the leftward and posterior QRS forces.” (Surawicz & Knilans, 2008). The so‐called voltage criteria,^1^ i.e., the increased QRS amplitude, are considered to be the specific finding for LVH.

**FIGURE 2:**
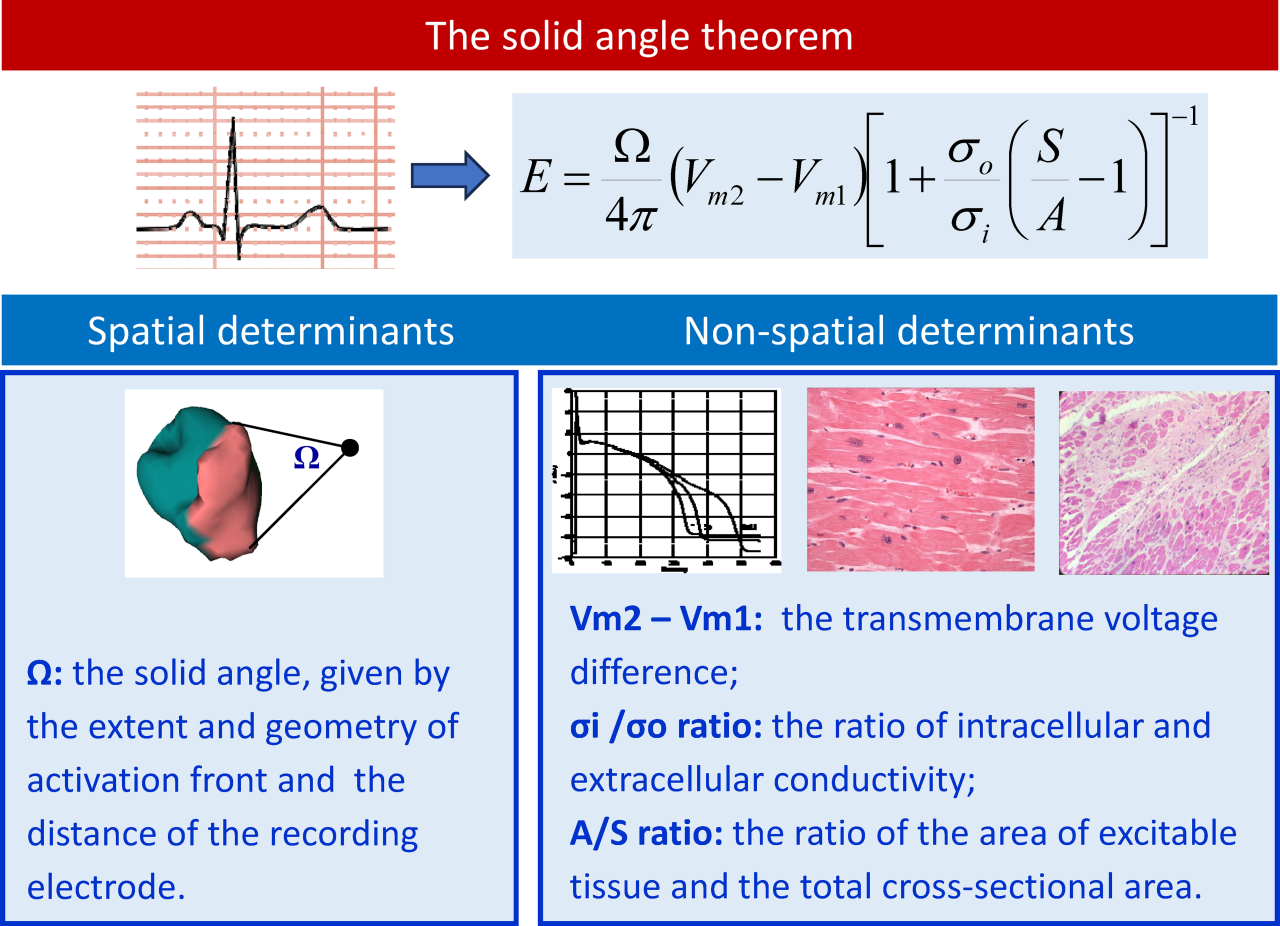
The spatial angle theorem (Holland & Arnsdorf, 1977; Holland & Arnsdorf, 1981) and the determinants influencing the QRS amplitude. The spatial angle theorem postulates that the externally recorded electrocardiographic potential is related to the spatial and non‐spatial determinants.

The theoretical background for the increased QRS amplitude I LVH is based on the *solid angle theorem* (Figure 2). In interpreting the changes in QRS amplitude according to the classical ECG‐LVH paradigm, the effect of the spatial factors is stressed, probably also due to the term “solid angle.” Stressing the spatial factors as the dominant primary factors shifted the attention away from the electrical properties of hypertrophied myocardium and the effort of electrocardiologists has focused on the best estimation of the LVM.

The ECG diagnosis of LVH (ECG‐LVH) is thus based on so‐called voltage criteria, i.e., the increased QRS amplitude (voltage). LVH is diagnosed when the QRS amplitude values exceed arbitrary upper normal limits. Till now a considerable number of ECG‐LVH criteria based on the increased QRS complex amplitude in either sole leads or combinations of leads have been recommended (Hancock et al., 2009). Therefore, there is a continuing effort to refine the voltage criteria to detect increased LVM.

The criteria basically followed the development in ECG, beginning with limb leads using later precordial leads, combining limb leads with precordial leads, and vectorcardiography. Later, Romhilt‐Estes criteria (Romhilt & Estes, 1968) turn the attention to additional ECG abnormalities, including P wave, QRS electrical axis, ST segment, QRS duration, and intrinsicoid deflection. However, the diagnostic performance has not fulfilled the expectations when compared to reference “non‐electrocardiographic” methods (Pewsner et al., 2007). Increased wall thickness and myocardial mass differ in their electrocardiographic manifestation (Maanja et al., 2020), which can contribute to the low diagnostic yield of ECG criteria for LVH. Given that clinical LVH (i.e., the increased LVM) is accompanied by a variety of underlying structural and electrophysiological changes, it is unlikely that under these conditions a direct proportionate relationship between LVM and QRS amplitude will occur. Importantly, diffuse myocardial fibrosis quantified as increased ECV by CMR has been shown to have an inverse effect on ECG QRS voltage amplitudes in LVH, and may thus obscure the ECG manifestations of LVH (Maanja et al., 2017). Moreover, CMR can be used to visualize and quantify focal myocardial fibrosis due to infarction or non‐ischemic scarring, myocardial disarray (Nielles‐Vallespin et al., 2017), and cardiomyocyte size (Ferreira de Souza et al., 2018). CMR is a versatile tool for interrogating and quantifying a number of pathophysiological processes related to LVH that have an important potential to be used to improve our understanding of the ECG.

## 8 ECG PATTERNS IN LVH OTHER THAN INCREASED QRS AMPLITUDE

Although the basic ECG‐LVH criterion is the increased QRS amplitude, the spectrum of QRS patterns in LVH is much more diverse. QRS patterns in patients with image‐confirmed LVH may also fall within normal limits (classically “false negative” results) and need explanation.

The QRS complex duration can be increased in LVH, and is included in some ECG‐LVH criteria, e.g., Romhilt‐Estes score, or Cornell voltage‐duration product. It is assumed that the QRS prolongation is due to the longer time to depolarize the larger myocardial mass. This assumption is logical; however, the computer simulations showed that the LVM is not the main determinant of the QRS duration, but the slowed impulse propagation considerably affects the QRS duration (Bacharova, Szathmary, Kovalcik, 2010). The prolongation of the QRS complex can result in patterns of intraventricular conduction disorders, including incomplete and even complete LBBBs. Left axis deviation (LAD) and left anterior fascicular block (LAFB) are common findings in LVH. In LAD, it is assumed that the amplitude of limb leads is affected by dilatation of the left ventricle to the left and posteriorly (Talbot, 1975). In the case of LAFB, the block in the anterior fascicle is assumed. However, the simulation studies showed comparable QRS patterns just by diffuse or localized slowing in CV in the LV myocardium (Bacharova et al., 2013; Bacharova et al., 2016). The underlying pathological changes in LV myocardium, especially fibrosis, can also result in fragmented QRS complexes (Kadi et al., 2013; Zhang et al., 2015) or even the presence of Q waves (Dohy et al., 2020).

The finding of LBBB on surface ECG in HF patients is very important since this defines candidacy for interventional treatment, e.g., CRT. Strauss pointed out that about one‐third of patients with LBBB are misdiagnosed, and the incomplete or complete LBBB patterns can be due to the combination of hypertrophy/dilatation and slowed CV (Strauss, 2012; Strauss et al., 2011; Upadhyay et al., 2019).

Simulation studies also support this assumption, showing the effect of the slowed CV on QRS patterns (Bacharova et al., 2012; Svehlikova et al., 2016). In this context, the term “LBBB” can be misleading, implicating just the conduction alteration in the conduction system, taking no account of the fact that the LBBB pattern could result from the slowed impulse propagation in the hypertrophied working myocardium, presumably even without a block in the left bundle. This fact is extremely important for cardiac resynchronization therapy (CRT) success, and it requires cardiologists' attention.

In summary, there are important QRS patterns beyond the increased QRS amplitude in patients with LVH that reflect important components of the underlying LV pathology.

Additionally, to QRS complex changes, there are primary and secondary repolarization changes (Bacharova, Szathmary, & Mateasik, 2010). The QT interval comprises both depolarization and repolarization. It follows that the QT interval prolongation in LVH can be due to both repolarization and depolarization alterations. The main underlying mechanisms are either CV slowing or action potential duration (APD) prolongation. The relative contribution of CV slowing and APD prolongation to prolonged QT duration, and QRS, ST, and T‐wave alterations, was demonstrated in an experimental model simulating the three types of anatomic LVH: concentric and eccentric hypertrophy, and left ventricular dilatation (Bacharova, Szathmary, & Mateasik, 2010).

The primary repolarization changes, simulated by APD prolongation, are characterized by a prolonged QT interval associated with modest QRS changes of amplitude and duration and notched or bifid T waves in left precordial leads of the 12‐lead ECG. The prolonged APD duration in LVH can be due to alterations of repolarization ion channels, due to genetic alterations or the effect of drugs, or volume overload.

The secondary repolarization changes related to CV slowing result in prolonged QT interval associated with major QRS alterations (increased QRS voltages, prolonged QRS duration, and changes in QRS morphology) and discordant tall T waves and a pattern of ST strain in the precordial leads. The decrease in CV can be related to alterations in density and distribution of gap junctions and of Cx43 expression and redistribution, observed already in the early stages of developing LVH. Additional contributing factors include fibrosis and alterations of sodium channels.

It is obvious that the ECG parameters and LVM provide complementary information on the status of the hypertrophied left ventricle. Regarding the false negative ECG results, analogous to the indexed LVM, using the ratio between QRS amplitude and LVM, i.e., the indexed/normalized QRS amplitude, the relation between QRS amplitude and LVM can be quantified. The QRS amplitude to the LV cross‐section/ LVM ratio normalizes the QRS amplitude on the LV cross‐section/LVM unit. This concept was introduced already in 1988 under the term “the specific potential of myocardium” (SP; Bacharova et al., 1989). It was shown in experimental LVH that the SP values were significantly lower as compared to controls (Bacharova et al., 2004, 2005) and were associated with a decreased expression of connexin43 (Bacharova et al., 2008). In clinical studies, the amplitude/mass ratio was inversely related to the extracellular volume measured by CMR (Maanja et al., 2017). Another possibility is to use the LVM to QRS ratio, as a diagnostic and prognostic measure (Porat et al., 2022; Slivnick et al., 2021).

## 9 ECG‐LVH AS AN INDEPENDENT CV RISK FACTOR

It is important to recognize that ECG‐LVH is an established electrophysiological marker with predictive properties of adverse prognosis independent of LV anatomy. Adam Leigh et al. (2016) compared the risk of CVD associated with ECG‐LVH and echo‐LVH in 4076 participants from the Cardiovascular Health Study (CHS), who were free of baseline CVD. ECG‐LVH. LVH diagnosed by ECG and echo were associated with an increased risk for CVD events adjusted for common CVD risk factors. The association between ECG‐LVH and CVD events was not substantively altered with further adjustment for echo‐LVH. In CHS, more coronary heart disease (CAD) events were identified in those with LVH at baseline compared to those without LVH (Drazner et al., 2004).

A meta‐analysis of 41,870 hypertensive patients [3] demonstrated that LVH detected by the Cornell voltage and Sokolow–Lyon criteria could independently predict the major cardiovascular events in hypertensive patients. In the general population, another meta‐analysis reported that individuals with ECG‐LVH by the Cornell voltage and Sokolow‐Lyon criteria had a 1.87‐fold and 1.66‐fold increased all‐cause mortality and cardiovascular mortality, respectively (You et al., 2020).

ECG‐LVH is an important risk factor for stroke, especially in hypertensive patients. Verdecchia et al. (2001) reported that ECG‐LVH may predict stroke, independent of BP and other CV risk factors. Zhao et al. (2020) recognized ECG‐LVH, defined by Sokolow‐Lyon criteria, as a strong predictor for stroke in hypertensive patients, especially those younger than 65 years. ECG‐LVH was strictly related to an increased risk of stroke after adjustment of both baseline SBP and mean SBP over the treatment period (Zhao et al., 2020). In a recent meta‐analysis including 58,098 hypertensive patients, patients with baseline ECG‐LVH had a 1.63‐fold increased risk of stroke by Cornell voltage criteria, 1.41‐fold by Cornell product criteria, and 1.42‐fold by Sokolow–Lyon voltage criteria, respectively (Yi et al., 2020).

Left ventricular remodeling is related to fibrosis and extracellular matrix alteration, which might result in progressive systolic dysfunction. In the Framingham Heart Study, patients without cardiovascular disease (CVD) but with severe ECG‐LVH had three times higher risk of composite CVD outcomes compared to patients with CVD and mild ECG‐LVH (Levy, Salomon, Agostino, 1994). In the Losartan Intervention for Endpoint Reduction in Hypertension (LIFE) study, patients with severe ECG‐LVH faced 14% higher risk of a composite cardiovascular outcome compared to those with mild ECG‐LVH. Moreover, ECG‐LVH regression in response to losartan also related to improved clinical cardiovascular outcomes independent of blood pressure response (Dahlöf et al., 2002). This result highlights the importance of improving ECG‐LVH by targeting the renin‐angiotensin‐aldosterone system. It was also shown that individual components of the Romhilt‐Estes score are associated with different CVD outcomes, it can be assumed that they indicate different pathophysiological processes in hypertrophied myocardium (Estes et al., 2015).

ECG‐LVH can co‐exist with intraventricular conduction defects, like LBBB and LAFB. In the Framingham study, individuals with LBBB showed a significantly elevated risk of cardiovascular death (Schneider et al., 1979). Recent studies also highlighted LBBB as an independent predictor for sudden cardiac death (Rabkin et al., 1980). Among patients with suspected exercise testing results, LBBB was independently associated with a 50% increase in all‐cause mortality (Hesse et al., 2001). In patients with HF with reduced ejection fraction, the presence of LBBB was associated with increased mortality (Baldasseroni et al., 2002; Witt et al., 2016). In contrast, the low QRS amplitude in subjects with LVH is reported as a cardiovascular risk factor (Bacharova et al., 2015; Pelliccia et al., 2023).

Interestingly, in spite of the evidence that ECG‐LVH is a significant cardiovascular risk factor (Levy, Salomon, D’Agostino, et al., 1994), ECG‐LVH is not included in cardiovascular risk scores (Arnett et al., 2019; Garg et al., 2017; S.‐O. Working Group and E. C. risk Collaboration et al., 2021; S. Working Group and E. C. Risk Collaboration et al., 2021).

## 10 NEW PARADIGM AND THE SUPPORT FOR THE NEW PARADIGM

This writing group strongly advocates that the role of ECG is not to find the best criterion for the increased LVM estimation (Bacharova, Estes, et al., 2010); that is the task of imaging in the current era. By principle, ECG cannot measure LV size or estimate the mass. The role of ECG is to identify the underlying electrical processes and to contribute to predicting the increased cardiovascular risk. Also, expecting that a single parameter can completely describe LVH is not realistic. Each QRS and repolarization pattern gives a partial view of the underlying processes. Research needs to be focused on the implications of individual ECG changes for diagnostics and potentially focused therapy and prevention.

The QRS patterns in LVH result from the interplay of anatomical, structural and electrical characteristics of the hypertrophied LV (Figure 1). The most frequent QRS patterns are: the increased QRS amplitude, pseudo‐normal QRS pattern, left axis deviation, increased QRS duration, LAFB, and incomplete and complete LBBBs. Additional ECG findings include ST strain, T wave changes, and QT interval prolongation. The theoretical support is obvious from the complex application of the solid angle theorem (Holland & Arnsdorf, 1977; Holland & Arnsdorf, 1981) considering the no‐spatial determinants of recorded voltage (Figure 2).

There are at least two reasons why to study also other than QRS voltage ECG parameters including the so‐called “false negative results”: (1) they are associated with LVH, logically we need to understand their pathophysiological background; (2) they have the independent predictive ability – the baseline R‐E score as well as the increasing score over time (Estes et al., 2015).

## 11 KNOWLEDGE GAPS AND FUTURE RESEARCH

Clinical and epidemiological research is focused on studying mainly the spatial determinant of the QRS in terms of the solid angle theorem, i.e., the LVM. On the other hand, the basic research provides a solid knowledge of the non‐spatial determinants, i.e., the electrical characteristics of hypertrophied myocardium, (for review, see i.e., Bacharova, 2007). Thus, there are knowledge gaps between the clinical interpretation of ECG and accumulated knowledge from the basic research. Since LVM is not the main and only determinant of the QRS amplitude, these gaps have to be addressed with the aim of understanding better the underlying mechanisms of ventricular activation in hypertrophied myocardium, to include it in the clinical interpretation of ECG and consequently into clinical decision making and targeted therapy. It is vital to understand the underlying pathophysiological mechanisms in relation to the true positive results, the false negative and false positive results as well as to the additional QRS patterns observed in patients with LVH.

The role of ECG in LVH evaluation is facing both challenges and opportunities. The key requirement is the interdisciplinary approach. The basic research can provide detailed information on the myocardial structure and function on tissue, cellular, subcellular, and molecular levels. It can study depolarization and repolarization currents, the characteristics of conduction in the conduction system, cardiomyocytes, myocardium, and the electrical impulse propagation, its sequence, and patterns. Experiments can be designed to study different stages of LVH, different clinical entities—CMP, hypertension, valvular diseases, obesity, amyloidosis, etc.

Clinical research can benefit from utilizing complementary information both from ECG and imaging methods as complex/composite information. That concept means to analyze the whole spectrum of ECG findings, including vectorcardiography associated with the increased LVM in different clinical conditions and different stages of LVH, pathological as well as “physiological.” The intraventricular conduction alteration patterns associated with LVH need to be re‐evaluated since they can be caused just by the altered CV in hypertrophied myocardium. Understanding the pathophysiological background will improve our understanding of the relation of ECG patterns to ventricular arrhythmias and HF, potentially target therapy, and monitor its effect.

ECG/QRS patterns associated with LVH represent markers of altered electrical properties; they are directly related to ventricular arrhythmias as well as to HF if the electro‐mechanical coupling is affected. The combination of anatomical LVH (increased LVM, optimal by CMR) and ECG patterns associated with the increased LVM, such as high QRS amplitude, false negative results, left axis deviation, LAFB, incomplete and complete LBBB, QT interval prolongation, ST deviation is obviously a challenge for developing new cardiovascular risk scores.

Based on the updated data, future research can utilize sophisticated graphical tools for simulating and visualizing the electrical impulse propagation and its relation to the surface ECG patterns. Artificial intelligence offers promising possibilities. Although artificial intelligence and ECG‐based machine learning applications showed better discrimination of LVH compared to traditional ECG rules outperforming cardiologists' conventional methods (Khurshid et al., 2021; Kwon et al., 2020), these powerful tools can be used to contribute to understanding the pathophysiological background and the independent predictive value of the variety of QRS patterns. Another promising direction of research is genomics, bringing new insights into the genetic basis of cardiac electrical phenotypes (Mayosi et al., 2008; van der Harst et al., 2016). Genomics can contribute to our understanding of biological pathways controlling myocardial mass and thus may potentially help to identify novel therapeutic targets.

In the future, a multimodal approach combining digital ECG, genomics, and AI is likely to improve the complex evaluation of LVH, using ECG as the fundamental cardiovascular marker providing information on the electrical characteristics of the heart, an underused cardiovascular marker in LVH evaluation.

## CONFLICT OF INTEREST STATEMENT

Niraj Varma, Elsayed Z. Soliman, Christian Jons, Philippe Chevalier are Editorial Board members of Annals of Noninvasive Electrocardiology and co‐authors of this article. To minimize bias, they were excluded from all editorial decision‐making related to the acceptance of this article for publication.

## Data Availability

Data sharing not applicable to this article as no datasets were generated or analysed during the current study.

## Endnote

^1^
Two terms are used: “the voltage criteria” when referring to the ECG‐LVH criteria, and “QRS amplitude” when referring to the size of the QRS complex deflections.
